# Effect of Extrusion Ratio on Mechanical Behavior and Microstructure Evolution of 7003 Aluminum Alloy at High-Speed Impact

**DOI:** 10.3390/ma17174219

**Published:** 2024-08-26

**Authors:** Rui Xing, Pengcheng Guo

**Affiliations:** 1 Intelligent Manufacturing and Mechanical Engineering, Hunan Institute of Technology, Hengyang 421002, China; xrui0802@163.com; 2 College of Mechanical and Vehicle Engineering, Hunan University, Changsha 410082, China; 3 Research Institute of Hunan University in Chongqing, Hunan University, Chongqing 400044, China

**Keywords:** extrusion ratio, high-speed impact, 7003 aluminum alloy

## Abstract

The extrusion ratio (ER) is one of the most important factors affecting the service performance of aluminum profiles. In this study, the influence of ER on the mechanical behavior and microstructure evolution of 7003 aluminum alloy at high-speed impact with strain rates ranging from 700 s^−1^ to 1100 s^−1^ was investigated. The studied alloy with an ER of 56 formed coarse grain rings during the heat treatment. The microstructure of the alloys with ERs of 20 and 9 is relatively uniform. The results indicate that under high-speed impact, the mechanical response behavior of the 7003-T6 alloy with different ERs is different. For the alloy with an ER of 56, strain hardening is the main mechanism of plastic deformation. In contrast, a flow stress reduction occurs at middle deformation stage for the ones with ERs of 20 and 9 due to concentrated deformation, which is more significant in the alloy with an ER of 20. Under high-speed impact, the alloy with an ER of 56 undergoes uneven plastic deformation due to the presence of coarse grain rings. The deformation is mainly borne by the region of coarse grains near the edge, and the closer to the center, the smaller the deformation. The deformation of the alloys with ERs of 20 and 9 is relatively uniform, but exhibits localized concentrated deformation in the area near the edge. The significant plastic deformation within deformation band causes a local temperature rise, resulting in a slight decrease in flow stress after the peak. These results can provide reliable data support for the application of 7003 aluminum alloy in the vehicle body crash energy absorption structure.

## 1. Introduction

Aluminum alloys are widely used in automotive, aerospace, and defense industries for their high strength, low density, good formability, and other advantages [1,2,3]. Among them, the 7xxx series aluminum alloy is one of the main materials used in these fields [4,5]. The components serving in the above fields not only need to withstand quasi-static loads, but may also encounter impact loads. Therefore, to improve the safety and reliability, the performance of 7xxx series aluminum alloys under high-speed impact must be investigated.

Extrusion is a commonly used forming method for aluminum alloys, such as the collision beams of vehicles, aircraft bodies, and high-speed train bodies, which are formed using extrusion technology. The parameters used in extrusion determine the mechanical performance of the formed profile [6], including the extrusion ratio (ER) [7], extrusion speed [8], extrusion temperature [9], and so on. Among them, the ER is one of the most important parameters that determine the extruded microstructure [10,11], so it has been widely investigated. Guang et al. [12] studied the ER on the microstructural characteristics and tensile properties of the Mg-Sm-based alloy, showing that the higher the ER, the coarser the recrystallized grains, and the weaker the fiber texture. In addition, a high ER may slightly reduce the strength of the profiles, but effectively improve their plasticity. Zhao et al. [13] investigated the effect of the ER on the strengthening mechanisms of a Mg-2Gd-xMn alloy. The results show that the strength is enhanced with the decrease in ER, due to the fact that alloys with lower ERs have a smaller proportion of recrystallization, and most of the grains are in hard orientation, making it difficult for dislocation slip. However, for a Mg-Sn-Zn-Ca alloy, the ultimate tensile strength and elongation are improved simultaneously with the increase in the ER [14]. Mu et al. [15] conducted quasi-static tensile and compression experiments on Mg-5Zn-1Mn alloys with different ERs. The results indicate that an increase in the ER leads to texture weakening and grain refinement, which can effectively improve its mechanical properties and alleviate tension compression asymmetry. However, although a large number of studies have been conducted on the effect of the ER on material properties, most of the research focuses on the quasi-static deformation of magnesium alloys, and the relevant studies on high-speed impact are extremely limited, especially for aluminum alloys.

The microstructure is an important factor affecting high-speed impact mechanical behavior [16]. Kim et al. [17] studied the deformation behavior of aluminum alloys with different original microstructures under high-speed impact. The results indicate that an uneven microstructure leads to uneven plastic deformation and promotes the formation of adiabatic shear bands. Owolabi et al. [18] investigated the effect of particulate reinforcement on adiabatic shear behavior of 6061-T6 aluminum alloys under high-speed impact, showing that the sensitivity to strain localization increases after particle reinforcement, leading to adiabatic shear fracture. Tiamiyu et al. [19] studied the effect of second-phase particle size on the high-speed impact performance of AA 2017 aluminum alloys. The results indicate that alloys with larger second-phase particles have a lower sensitivity to strain localization. Gao et al. [20] investigated the evolution of precipitates in the 2519-T87 aluminum alloy during high-speed impact with strain rates ranging from 667 s^−1^ to 7050 s^−1^. The results show that the quantity and morphology of *θ*′ phases change significantly only when the applied strain rate is higher than 5730 s^−1^. The higher the strain rate, the smaller the quantity and aspect ratio of *θ*′ phase, which leads to a decrease in the microhardness. As mentioned above, the ER largely determines the microstructure of profiles, that is, the ER certainly affects the mechanical behavior and microstructure evolution of aluminum profiles under high-speed impact. However, the influence law is not clear, so it is necessary to carry out related research to provide reliable data support for the application of extruded profiles. 

As mentioned above, although the effect of the ER on the mechanical properties has been extensively studied, there are few studies on 7xxx series aluminum alloys, especially under high-speed impact loading. In this study, high-speed impact experiments were performed on 7003 aluminum alloy with various ERs at strain rate range of 700 s^−1^ to 1100 s^−1^. The dependence of mechanical response and microstructural evolution on the ER under high-speed impact was studied.

## 2. Experimental Procedure

The actual measured composition of the studied 7003 aluminum alloy is shown in Table 1. Extrusion profiles with ERs of 56, 20, and 9 were obtained by designing solid round bar dies with extrusion outlet diameters of 12 mm, 20 mm, and 30 mm using extrusion billets with a diameter of 90 mm. The extrusion parameters are shown in Table 2. The extruded round bars were immediately cooled to room temperature after leaving the mold.

**Figure 1:**
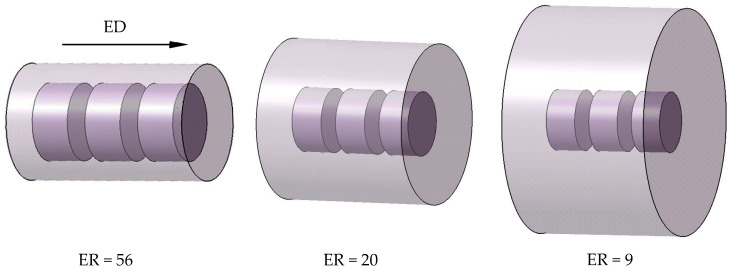
Schematic of sampling from extruded bars with different ERs.

Cylindrical specimens with diameter of 8 mm and height of 4 mm were cut from the extrusion bars using a wire-cut electrical discharge machine. To ensure uniformity, all the specimens were taken from the center of bars along the extrusion direction (ED). The sampling positions on bars with different ERs are shown in Figure 1. To better reflect the actual service of 7003 aluminum alloy profiles, all the cylindrical specimens were solution treated at 470 °C for 30 min and then immediately cooled to room temperature with water, followed by aging at 120 °C for 12 h. The heat treatment process used here is referred to as T6.

All the high-speed impact experiments with strain rates in the range of 700 s^−1^ to 1100 s^−1^ were carried out using a split Hopkinson pressure bar (SHPB) device (Baisen Experimental Equipment Co., LTD, Changsha, China) along ED at room temperature. After impact experiments, the specimens were immediately cooled to room temperature with water to preserve the deformation microstructure. Each strain rate was repeated at least three times to ensure the accuracy of the obtained data.

The SHPB experiments are based on one-dimensional stress wave theory and uniformity assumption. Under these conditions, the stress, strain, and strain rate of the specimen during impact can be calculated by the following equation [21]:

(1) $$\varepsilon =-2\frac{C_{0}}{L_{0}}\int_{0}^{t}\varepsilon_{r}dt$$

(2) $$\dot{\varepsilon }=-2\frac{C_{0}}{L_{0}}\varepsilon_{r}$$

(3) $$\sigma =\frac{A}{A_{s}}E\varepsilon_{t}$$

where *C*_0_ is the propagation velocity of the wave in the pressure bar, *L*_0_ is the initial length of the specimen, $\varepsilon_{r}$ is the strain history of reflected incident bar, *A* and *A_s_* are the cross-sectional areas of the SHPB bars and specimen, respectively, *E* is the elastic modulus of the pressure bar, $\varepsilon_{t}$ is the strain history of transmitted bar, and *t* is the deformation time of specimen.

The specimens before and after deformation were cut using wire-electrode cutting through a plane passing through axis of cylinder. Cutting surfaces were ground sequentially using 800, 1000, 2000, and 5000 grit SiC papers and subsequently polished until the surface was free of visible scratches. The texture of specimens was measured using X-ray diffraction (XRD) technology. For optical microscope (OM) observation, the polished surfaces were etched with an acid solution of 2.5% HBF_4_-97.5% H_2_O (Runfeng Petrochemical Co., Ltd., Nantong, China). For transmission electron microscope (TEM) observation, specimen with thickness of around 0.5 mm was cut along the cylindrical axis. Then, obtain disk-shaped slices with diameter of 3 mm and thickness of 0.1 mm from it. Subsequently, these slices were thinned using an ion thinning instrument. A Tecnai G2 20 TEM, engineered by FEI Company, Hillsboro, OR, USA, was used to observe the samples at voltage of 200 kV.

**Figure 2:**
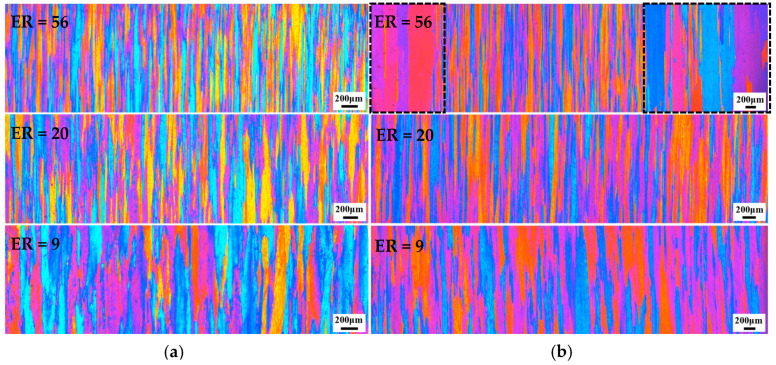
OM images of the 7003 aluminum profile with different ERs: (**a**) as-extruded state; (**b**) T6 treated state.

OM images of the 7003 aluminum alloy in the as-extruded and T6 states are shown in Figure 2. The grain size statistics are shown in Table 3. The microstructure of the as-extruded specimens with ERs of 56, 20, and 9 is composed of elongated fibrous grains with average grain sizes of 27 μm, 64 μm, and 110 μm, respectively. After T6 treatment, partial recrystallization occurred inside the specimen with ER of 56 to form a coarse grain ring, resulting in inhomogeneous microstructure. As indicated by the dashed box in the Figure 2b, the grains located at the edge of cylinders are apparently coarse, with an average width of about 221 μm. In contrast, the fiber grain located in the center is slender, with an average width of about 29 μm. For the alloys with ERs of 20 and 9, the microstructure after T6 treatment is still the same elongated fibrous grains as before, that is, recrystallization does not occur.

## 3. Results

### 3.1. Macroscopic Morphology

**Figure 3:**
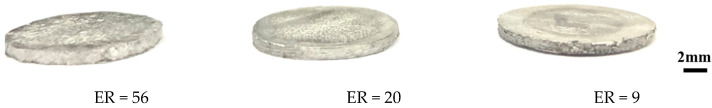
Macroscopic morphologies of the impacted 7003-T6 specimens with different ERs at strain rate of 900 s^−1^.

**Figure 4:**
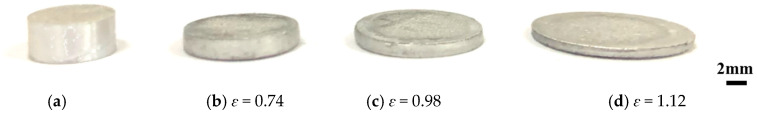
Comparison of macroscopic morphologies of the 7003-T6 specimens with ER of 20: (**a**) undeformed state; (**b**) 700 s^−1^; (**c**) 900 s^−1^; (**d**) 1100 s^−1^.

The macroscopic morphologies of the 7003-T6 profile before and after deformation are shown in Figure 3 and Figure 4. It can be seen that after high-speed impact deformation, all specimens did not exhibit fracture failure. Moreover, the end faces of the deformed specimens remain nearly circular, indicating relatively uniform plastic deformation. At the same strain rate, the differences in morphology and height of the deformed specimens with different ERs are small, as shown in Figure 3. As shown in Figure 4, when impacted at strain rate of 700 s^−1^, the specimen height is reduced by ~52% to a true strain of 0.74. As the impacted strain rate increases to 900 s^−1^, the height is reduced by ~62% with a true strain of 0.98. The height reduction is ~67% with a true strain of 1.12 when the impacted strain rate further increases to 1100 s^−1^.

### 3.2. Stress Response Behavior

**Figure 5:**
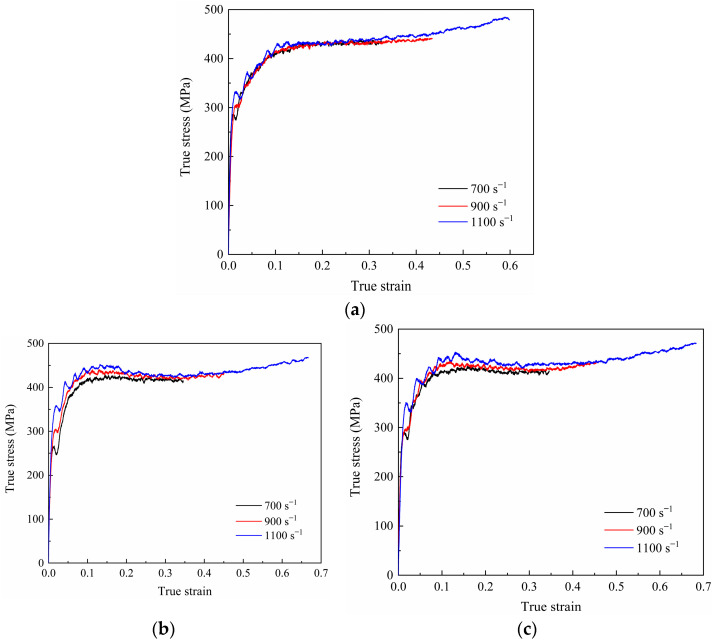
True stress–true strain curves of the 7003-T6 alloy with ERs of (**a**) 56, (**b**) 20, and (**c**) 9.

**Figure 6:**
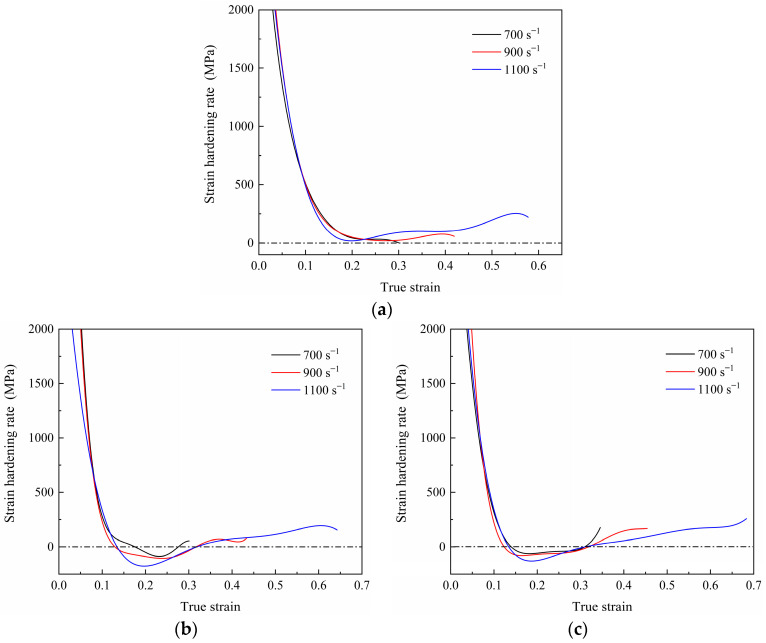
SHR curves of specimens with ERs of (**a**) 56; (**b**) 20; (**c**) 9.

The true stress–true strain curves of the 7003-T6 alloy with different ERs at strain rates of 700 s^−1^, 900 s^−1^, and 1100 s^−1^ are shown in Figure 5. Clearly, there are many burrs on the true stress–true strain curves, so they cannot be directly used for subsequent calculations. The polynomial fitting method is used to smooth the above curves. Here, these curves are fitted using a seventh-order polynomial [22]. The strain-hardening rate (SHR) curve is obtained by solving the first-order derivative of the fitted curve, as shown in Figure 6.

For the alloy with an ER of 56, the flow stress at different strain rates shows a similar trend and is not sensitive to the applied strain rate. As shown in Figure 5a and Figure 6a, after elastic deformation, the specimen undergoes plastic deformation. In the initial stage of the plastic deformation, the flow stress increases with the applied strain due to dislocation multiplication and accumulation, showing a very high strain-hardening ability. Subsequently, dynamic recovery occurs within the material, and the interaction and annihilation of dislocations lead to a gradual decrease in the SHR to near 0, implying a gradual equilibrium between strain hardening and dynamic recovery [23]. Interestingly, at strain rates of 900 s^−1^ and 1100 s^−1^, the flow stress slowly increases with strain again after the strain increases to around 0.26 and 0.20, respectively, indicating that the strain-hardening behavior once again dominates the plastic deformation. In addition, it can be observed that there are significant fluctuations in the flow stress curve at a strain rate of 1100 s^−1^, which is reported to be caused by impedance mismatch between the contact surface of specimen and SHPB [24].

Similarly, for the alloy with an ER of 20, at a strain rate of 700 s^−1^, in the initial stage of plastic deformation after the elastic stage, the flow stress increases with strain, but the SHR gradually decreases. Until the impacted strain increases to approximately 0.17, the flow stress reaches its peak. Then, the flow stress slightly decreases until the strain increases to about 0.27, and the SHR reaches 0 again. After that, the flow stress remains essentially stable. Accordingly, plastic deformation during high-speed impact deformation is the result of the combined action of strain hardening and thermal softening [24]. Temperature rise promotes dislocation motion and dislocation annihilation, resulting in a decrease in dislocation density. In contrast, the strain-hardening effect leads to an increase in dislocation density. The effects of both reach a balance so that the flow stresses are stabilized [19]. At strain rates of 900 s^−1^ and 1100 s^−1^, the strains required to reach their stress peaks are small, around 0.13. After the peak, the SHR becomes negative, and decreases then increases with the increase in impacted strain, that is, the flow stress decreases slowly at first and then increases again. When the impacted strain increases to around 0.32, the SHR reaches 0 again. Afterwards, the flow stress gradually increases with impacted strain, that is, strain hardening dominates the plastic deformation again.

For the specimen with an ER of 9, the trends in the true stress–true strain curves at various strain rates are similar to those of the specimen with an ER of 20. The most obvious difference is that, at strain rates of 700 s^−1^ and 900 s^−1^, the stress reduction after the peak stress is relatively small. At a strain rate of 1100 s^−1^, compared to low strain rates, the stress reduction after peak stress becomes significant. The flow stress reaches its peak at a true strain of around 0.13. Subsequently, it decreases with strain until the true strain increases to about 0.31. At this point, strain hardening and dynamic recovery once again reach a basic equilibrium. Then, as the impacted strain continues to increase, the flow stress increases slowly, which has never been observed under quasi-static loads.

**Figure 7:**
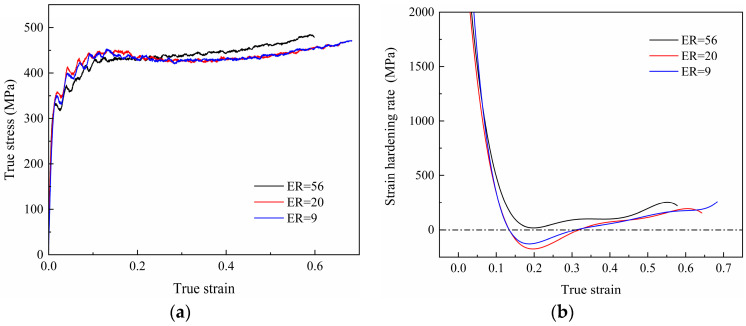
(**a**) Flow stress and (**b**) SHR of specimens with different ERs at strain rate of 1100 s^−1^.

At a strain rate of 1100 s^−1^, the true stress—strain curves and SHR curves of specimens with different ERs are shown in Figure 7. Clearly, the mechanical response behavior of specimens with various ERs is different. For the specimen with an ER of 56, strain-hardening behavior is the main deformation behavior. For specimens with ERs of 20 and 9, flow stress reduction occurs during deformation, which is more significant in the alloy with an ER of 20.

### 3.3. Deformed Microstructure 

**Figure 8:**
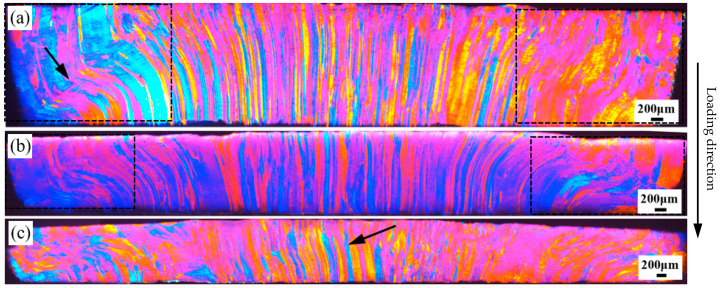
Deformed OM images of the 7003-T6 alloy with ER of 56 at strain rates of (**a**) 700 s^−1^, (**b**) 900 s^−1^, and (**c**) 1100 s^−1^, corresponding to true strains of 0.74, 0.98 and 1.12, respectively.

**Figure 9:**
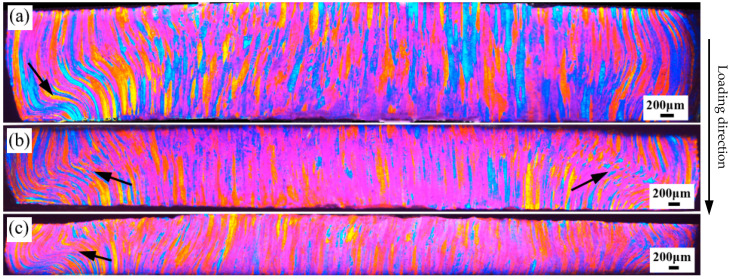
Deformed OM images of the 7003-T6 alloy with ER of 20 at strain rates of (**a**) 700 s^−1^, (**b**) 900 s^−1^, and (**c**) 1100 s^−1^, corresponding to true strains of 0.74, 0.98, and 1.12, respectively.

**Figure 10:**
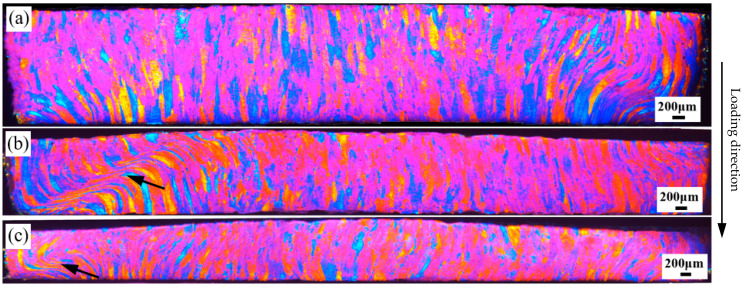
Deformed OM images of the 7003-T6 alloy with ER of 9 at strain rates of (**a**) 700 s^−1^, (**b**) 900 s^−1^, and (**c**) 1100 s^−1^, corresponding to true strains of 0.74, 0.98, and 1.12, respectively.

Deformed OM images of the 7003-T6 alloy with different ERs are shown in Figure 8, Figure 9 and Figure 10. All the specimens were subjected to free impact at strain rates of 700 s^−1^, 900 s^−1^, and 1100 s^−1^, with true strains of 0.74, 0.98, and 1.12, respectively. These images represent the entire observation surface.

As shown in Figure 8, for the specimen with an ER of 56, grains distorted to an S-shape are observed on the left side when impacted at a strain rate of 700 s^−1^, which have no obvious boundary with the matrix. Interestingly, most of the grains that undergo torsional deformation are coarse grains near the edge of specimen. In contrast, the average width of the grains near the center of specimen is smaller, and no significant torsional deformation is detected due to a visibly lower degree of deformation, as shown in Figure 8a. When deformed at a strain rate of 900 s^−1^, similar to the low strain rate, the grains near the edge of the specimen undergo torsional deformation, but no obvious deformation bands are observed, indicating relatively uniform plastic deformation in the region. The grains at the center of the specimen still maintain a fibrous shape parallel to the loading direction, and the grain boundaries are clearly distinguishable, indicating that the grain deformation is relatively small. When the strain rate increases to 1100 s^−1^, almost all grains on the observation surface undergo deformation. Among these, grains near the edge of the specimen exhibit relatively large deformation, and their grain boundaries are difficult to identify. In contrast, the grains at the center of the specimen exhibit relatively small deformations, and their grain boundaries are clearly distinguishable. As indicated by the arrow in Figure 8c, the initial fibrous microstructure can still be observed at the center of the specimen.

Deformed OM images of the 7003-T6 alloy with an ER of 20 after high-speed impact are shown in Figure 9. At a strain rate of 700 s^−1^, the plastic deformation is relatively uniform compared to the results of the alloy with an ER of 56. Almost all grains on the observation surface undergo significant deformation, but the deformation characteristics are different in different regions. Concentrated deformation is observed close to the edges of the specimen, while the deformation in the center is more uniform. As shown in Figure 9a, a deformation band is observed near the edge on the left side of the OM image (indicated by the arrow), where the grains are twisted and deformed into an S-shape. In contrast, the grains at the center of the specimen do not undergo torsional deformation, but their grain boundaries are difficult to distinguish, indicating significant deformation. When the strain rate is 900 s^−1^, as indicated by the arrows in Figure 9b, deformation bands are observed near the edges of the specimen on both sides of the OM image. Compared with low strain rates, the width of the deformation bands is larger. The grains outside the deformation band are fibrous and have clearly distinguishable grain boundaries, indicating relatively small deformation. Relatively, the deformation near the center of the specimen is more uniform, and it is challenging to identify the grain boundaries, demonstrating significant deformation of the grains. When the strain rate is 1100 s^−1^, similar to low strain rates, the plastic deformation at the edges of the specimen is mainly concentrated in the deformation band, as indicated by the arrow in Figure 9c, while the deformation in the center is relatively uniform. The grain boundaries near the edge of the specimen are relatively clear, while those in the center are indistinguishable, indicating that the deformation at the center is greater compared to the edge of the specimen.

For the alloy with an ER of 9, after deformation at a strain rate of 700 s^−1^, twisted grains are observed on the right side of the OM image. As shown in Figure 10a, the grain boundary in the center of the specimen is difficult to distinguish, indicating that there is a large deformation in this region. When the strain rate is 900 s^−1^, a deformation band that develops almost through the upper and lower surfaces of the specimen is observed at the position indicated by the arrow on the left side of the OM image shown in Figure 10b. The band is at an angle of about 60° to the loading direction, and the grains in the band are violently stretched into extremely fine fibrous shapes. For the matrix below the deformation band, the boundaries of the grains are still clearly recognizable, indicating less plastic deformation. Comparatively, above the deformation band, the boundaries of some grains become blurred, implying significant deformation. In contrast, the deformation in the region outside the deformation band is relatively uniform. When the strain rate increases to 1100 s^−1^, as shown in Figure 10c, the plastic deformation of the entire specimen is relatively uniform, with concentrated deformation observed only in a small region indicated by the arrow near the edge of the specimen.

In summary, under high-speed impact, the alloy with an ER of 56 experiences inhomogeneous plastic deformation, while the alloys with ERs of 20 and 9 undergo relatively homogeneous deformation, but there is concentrated deformation near the edges. In comparison, the concentrated deformation is more significant in the alloy with an ER of 20.

## 4. Discussion

### 4.1. Influence of ER on Microstructure

As shown in Figure 2, the microstructure of the 7003-T6 alloy with an ER of 56 is uneven due to partial recrystallization, with coarse grains at the edges and grains of smaller width at the center. While the microstructure distribution of the specimens with ERs of 20 and 9 is uniform, consisting of fibrous grains with similar width and parallel to the ED. The uneven microstructure may be the reason for the inhomogeneous plastic deformation of the specimen with an ER of 56 under high-speed impact. The fact that the specimen with an ER of 56 recrystallized during heat treatment, while other specimens did not, can be attributed to the different degree of deformation of the alloys during the extrusion molding process [23,25].

**Figure 11:**
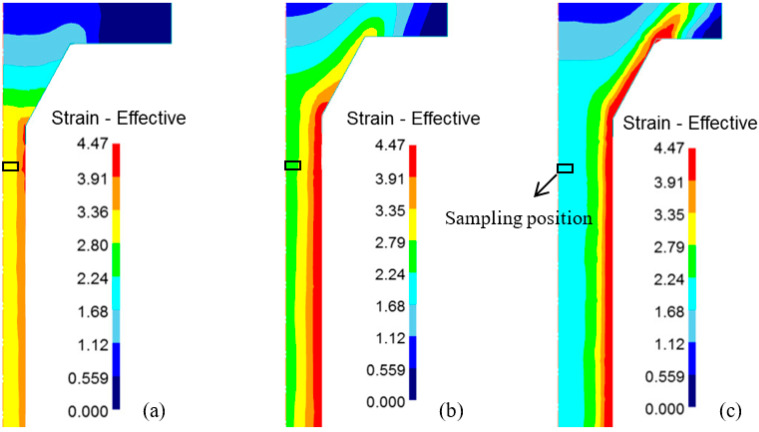
Distribution of effective plastic strain of the 7003 alloy with ER of (**a**) 56, (**b**) 20, and (**c**) 9.

The ER directly reflects the amount of deformation of the metal in the extrusion process, the larger the ER, the greater the degree of deformation. Here, finite element models were established using DEFORM 11.0 software to simulate the extrusion process of the alloy. According to the modeling method reported in the literature [26], the extrusion cylinder, dummy block, 7003 aluminum alloy billet, and mold are established in the DEFORM software. Subsequently, the relative motion relationship between these components is established. The molding process of bars with different ERs is simulated according to the parameters shown in Table 2. Figure 11 shows the strain distribution during the molding process of bars with different ERs simulated using DEFORM software. It can be seen that the strain distribution in the outer layer of the extruded alloy is not uniform, and the closer to the surface layer, the higher the strain. In contrast, the deformation in the center of the extruded alloy is relatively uniform. It is worth noting that the larger the ER, the smaller the diameter of the extruded bar, and the smaller the diameter of the uniform plastic deformation zone. In this study, in order to maintain uniformity, specimens were taken from the center of extruded bars. The sampling locations are shown as boxed locations in Figure 11. It can be seen that the specimen with an ER of 56 has the greatest overall deformation compared to the other specimens, among which the strain at the edge of the specimen is greater because it is close to the surface inhomogeneous deformation region of the extruded bar. However, for specimens with ERs of 20 and 9, the sampling edges are far away from the uneven deformation region of the extrusion bar, especially the specimen with an ER of 9, which is completely within the uniform deformation area of the extrusion bar. The simulation results show that the deformation degree of these specimens is much smaller, compared to the specimens with an ER of 56.

**Figure 12:**
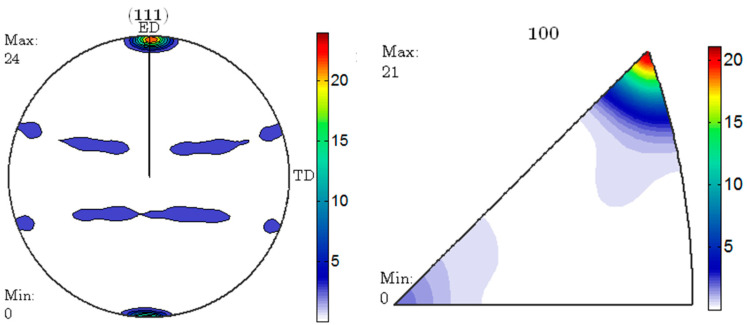
Polar and inverse pole diagrams of the as-extruded 7003 alloy with ER of 56.

**Figure 13:**
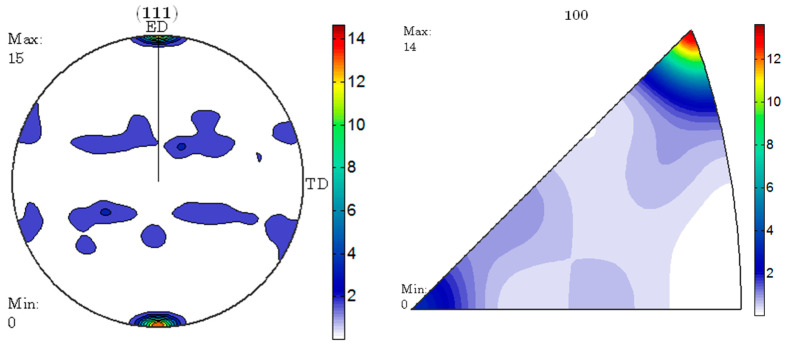
Polar and inverse pole diagrams of the 7003-T6 alloy with ER of 56.

According to reports, there is a close relationship between the degree of deformation and the static recrystallization temperature. The greater the degree of deformation, the lower the static recrystallization temperature [23,25]. This is because larger plastic deformation causes lattice distortion, resulting in a large number of dislocations and internal stresses. In this case, the internal energy storage of the metal is relatively high, so it is in a thermodynamically unstable high free energy state. The greater the degree of plastic deformation, the higher the stored energy inside the metal. Storage energy is the driving force for static recrystallization, and the larger the storage energy, the greater the driving force, and the lower the recrystallization temperature [27,28]. Among the three groups of specimens, the specimen with an ER of 56 has the highest deformation and the highest deformation storage energy. Therefore, compared with other specimens, this group of specimens has the lowest recrystallization temperature. For this reason, only the specimen with an ER of 56 is recrystallized under the same heat treatment process, while the other specimens do not undergo recrystallization. For the specimen with an ER of 56, the deformation at the edges is relatively large and uneven. So, recrystallization starts at the edges of the specimen and gradually develops towards the center. This is the reason why the specimen with an ER of 56 after T6 treatment has coarse grains at the edges and smaller grains in the center. The pole and reverse pole diagrams of the specimen with an ER of 56 before and after T6 treatment are shown in Figure 12 and Figure 13. It can be seen that the extrusion texture strength of the specimen significantly decreases due to recrystallization.

### 4.2. Influence of Mcrostructure on Plastic Deformation

**Figure 14:**
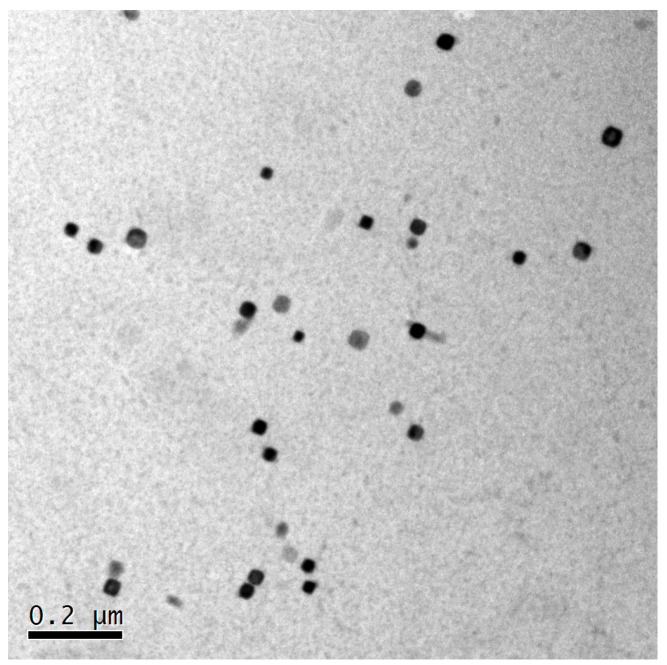
TEM image of the 7003-T6 alloy with ER of 56.

According to reports [10,11,12,13], the ER has a significant impact on the microstructure morphology, manifesting a considerable difference in grain size between alloys with different ERs. In this study, the 7003 aluminum profiles with different ERs were T6 treated, which reduced the effect of dislocations and precipitation on deformation. As shown in Figure 14, the dislocation density in the 7003-T6 alloy is very low, while a uniformly distributed precipitated phase is produced. In summary, for the studied 7003 alloy, the grain morphology is the most important factor leading to differences in plastic deformation under high-speed impact.

Dislocation motion is an important mechanism of plastic deformation in aluminum alloys, including the slip, climb, and interaction of dislocations. Among them, dislocation slip is the main mode of motion for dislocations [29]. Grain boundaries are the main obstacle to dislocation motion. Because the irregular arrangement of atoms on grain boundaries is different from the periodic arrangement inside grains, this requires dislocations to have higher energy to pass through grain boundaries [23,30]. Therefore, the larger the grain size, the fewer grain boundaries in the same volume, the fewer obstacles to dislocation movement, and the more easily plastic deformation occurs.

For the specimen with an ER of 56, the grain size distribution is uneven, with coarse grains near the edges of the specimen and smaller widths of fibrous grains in the center. In this case, large grains near the edges are more prone to plastic deformation, while plastic deformation in the center region of the specimen is relatively difficult. Therefore, the plastic deformation of the specimen is uneven, mainly occurring in areas with coarse grains. In addition, during high-speed impact deformation, the velocity of dislocation motion significantly increases, leading to an increase in short-range resistance, which results in an increase in the deformation resistance of the material [31,32]. Therefore, during high-speed impact deformation, the ability of grain boundaries to hinder dislocation motion is enhanced, further exacerbating the plastic deformation non-uniformity caused by uneven grain size.

For specimens with ERs of 20 and 9, the grain size does not change after T6 treatment, and the microstructure is relatively uniform. Therefore, compared with the specimen with an ER of 56, the plastic deformation is more uniform. Significant plastic deformation occurs in all regions of the specimen, with localized concentrated deformation near the edges and uniform deformation at the center of the specimen. At strain rate of 1100 s^−1^, the localized concentrated deformation phenomenon is more significant in the specimen with an ER of 20 compared to the specimen with an ER of 9. This can be attributed to the smaller grain size of the specimen with an ER of 20. According to reports, during plastic deformation, dislocation plugging occurs when dislocations move near grain boundaries, causing concentrations of stress and strain [30,33]. Therefore, the smaller the average width of fibrous grains, the larger the proportion of grain boundaries with the same volume, which enhances the hindering effect of grain boundaries on dislocation movement and makes the strain concentration phenomenon more significant in the specimen. In addition, during high-speed impact deformation, the deformation resistance is enhanced, further promoting strain concentration. The combined effect of the two causes plastic deformation to concentrate within the deformation band.

Under high-speed impact, the flow stress slightly decreased after reaching its peak, which can be attributed to local concentrated deformation. According to reports, during high-speed impact deformation, due to the fast deformation speed and short deformation time, the heat converted from plastic deformation cannot diffuse in time, resulting in an increase in the temperature of the specimen [34,35]. Temperature increases promote dislocation motion and dislocation annihilation, which leads to a decrease in flow stresses. Especially, the strain within the deformation zone is relatively large. The heat converted from plastic work is concentrated within the deformation band, resulting in a higher temperature in the band compared to other regions. The temperature rise $T_{*}$ within the deformation band can be estimated by the following equation [24]:

(4) $$T_{*}=\frac{0.9\tau_{*}\gamma_{*}}{\rho c}$$

where *ρ* and *c* are the density and heat capacity of the studied 7003 aluminum alloy, which are 2.7 × 10^3^ kg/m^3^ and 875 J/(kg·K), respectively. $\tau_{*}$ is shear stress in deformation band, which can be estimated using flow stress. $\gamma_{*}$ is shear strain in band, which can be estimated by the ratio of the displacement of deformed grains along the shear direction to the width of the deformation band [36]. For the specimen with an ER of 20, at a strain rate of 1100 s^−1^, the relevant parameters are $\tau_{*}$ = 449 MPa and $\gamma_{*}$ = 0.72. According to Equation (4), the estimated temperature rise within the deformation band is around 123 °C, so the temperature within the band is approximately 148 °C. The temperature rise causes the deformation in this region to exhibit softening, which results in a slight decrease in the flow stress. As the strain increases, strain hardening due to deformation in other regions once again dominates plastic deformation, resulting in a gradual increase in flow stress with strain.

## 5. Conclusions

The influence of ER on the mechanical behavior and microstructure evolution of 7003-T6 alloy at strain rates of 700 s^−1^ to 1100 s^−1^ was studied. The main conclusions drawn are as follows:

1. The microstructure shows significant differences in 7003-T6 alloys with various ERs. The larger ER resulted in partial recrystallization and formation of coarse grain ring in the alloy with an ER of 56 after T6 treatment, leading to an inhomogeneous microstructure. Due to the absence of recrystallization, the microstructure of the alloys with ERs of 20 and 9 is homogeneous, consisting of fibrous grains with average widths of 66 μm and 122 μm, respectively.
2. Due to the different initial microstructures, the mechanical behavior of the 7003-T6 alloy with various ERs exhibits obvious differences at strain rates ranging from 700 s^−1^ to 1100 s^−1^. For the alloy with an ER of 56, strain hardening is the main deformation behavior. For ones with ERs of 20 and 9, the flow stress slightly decreased after the peak stress, and then exhibited strain hardening again as the applied strain increased.
3. At strain rates ranging from 700 s^−1^ to 1100 s^−1^, the plastic deformation of the alloy with an ER of 56 is uneven, with larger deformation concentrated in the coarse grain at the edges and smaller deformation at the center with slender grains. The plastic deformation of the alloys with ERs of 20 and 9 is relatively homogeneous, while the center and edges exhibit different features, with concentrated deformation occurring near the edge and uniform deformation occurring in the center. In comparison, the concentrated deformation is more significant in the alloy with an ER of 20.

## Figures and Tables

**Table 1:** Actual measured chemical composition (wt-%) of the 7003 aluminum alloy.

Zn	Mg	Cu	Fe	Si	Mn	Zr	Cr	Ti	V	Al
5.87	0.70	0.06	0.14	0.05	0.13	0.15	0.07	0.03	0.01	Bal.

**Table 2:** Extrusion experiment parameters of the 7003 aluminum alloy.

Extrusion Speed (mm s^−1^)	Billet Temperature (°C)	Die Temperature (°C)	Container Temperature (°C)	ER
1	450	450	430	56/20/9

**Table 3:** Grain size of the 7003 aluminum profile with different ERs in as-extruded and T6 treated states.

ER	As-Extruded State	T6 Treated State
56	27 µm	center: 29 µm edge: 221 µm
20	64 µm	66 µm
9	110 µm	112 µm

## Data Availability

The original contributions presented in the study are included in the article, further inquiries can be directed to the corresponding author.
